# Oral Islatravir in Macaques Decreases Lymphocytes and Monocytes and Is Associated with Immune Alterations

**DOI:** 10.3390/pharmaceutics18030381

**Published:** 2026-03-20

**Authors:** Michele B. Daly, Daniel Kim, Seidu Inusah, Dawn Little, Jiyoung S. Kim, Natalia Makarova, Tiancheng E. Edwards, James Mitchell, Walid Heneine, Yi Pan, Charles W. Dobard, J. Gerardo García-Lerma

**Affiliations:** 1 Laboratory Branch, Division of HIV Prevention, National Center for HIV, Viral Hepatitis, STD, and TB Prevention, Centers for Disease Control and Prevention, Atlanta, GA 30333, USA; 2 Quantitative Sciences Branch, Division of HIV Prevention, National Center for HIV, Viral Hepatitis, STD, and TB Prevention, Centers for Disease Control and Prevention, Atlanta, GA 30333, USA

**Keywords:** islatravir, lymphopenia, nonhuman primate model, preclinical safety, flow cytometry

## Abstract

**Background:** Islatravir (ISL) is a first-in-class nucleoside reverse transcriptase translocation inhibitor with high potency and long half-life in peripheral blood mononuclear cells (PBMCs). However, treatment and prevention of HIV with oral ISL in humans has been associated with decreases in total lymphocytes, CD4 T-cells, and B-cells in a dose-dependent manner. We investigated in macaques the effects of oral ISL on lymphocytes, monocytes, granulocytes, and gene expression in PBMCs. **Methods:** Female pig-tailed macaques (*n* = 5) received an HIV pre-exposure prophylaxis dose of oral ISL adjusted allometrically once a week for 12 weeks. Complete blood counts and B- and T-cells were monitored prior to, during, and after ISL treatment, and changes in counts were evaluated by using a repeated measures model. Changes in gene expression were investigated in PBMCs during treatment and following treatment discontinuation. **Results:** ISL treatment was associated with declines in lymphocytes (11.9%, *p* = 0.0015) and monocytes (22.4%, *p* = 0.0003), but not granulocytes (0.3%, *p* = 0.9781). Total lymphocytes and monocytes returned to pre-treatment levels 6 weeks after treatment cessation (*p* = 0.8244 and *p* = 0.4620, respectively). Lymphocyte subpopulation analyses showed a significant decline in CD8 (−18.4%, *p* = 0.0364) and CD20 (−35.3%; *p* = 0.0002) cells but not CD4 cells (−7.4%; *p* = 0.3470). Gene set enrichment analysis showed negative enrichment (p_adj_ < 0.05) of gene pathways associated with immune regulation, cell proliferation, and inflammation. **Conclusions:** ISL treatment resulted in significant reductions in lymphocytes reproducing clinical toxicity. This effect was reversed after treatment cessation as observed in humans. Our results highlight the value of the macaque model to study immune alterations at the preclinical stage.

## 1. Introduction

Islatravir (ISL) is a first-in-class nucleoside reverse transcriptase translocation inhibitor (NRTTI) with promising characteristics for HIV treatment and prevention. ISL is highly potent against wild-type and drug-resistant HIV, and its intracellular active form (ISL-triphosphate, ISL-TP) has a long half-life in peripheral blood mononuclear cells (PBMCs) [1]. The high potency and long half-life of ISL-TP prompted the evaluation of multiple ISL dosing strategies in humans for HIV treatment or pre-exposure prophylaxis (PrEP), including daily, weekly, and monthly regimens [2,3,4,5]. However, the development of ISL experienced setbacks in late 2021 when several trials were paused due to declines in total lymphocytes, CD4 T-cells, and B-cell counts. Exposure-related reductions in lymphocytes were observed with daily ISL doses exceeding 0.75 mg and with monthly PrEP doses of 60 mg and 120 mg [6,7]. These observations led to safety reassessments and dose optimization efforts to identify safe and effective doses of ISL for further clinical development.

The precise mechanisms underlying ISL-induced lymphopenia are not known but are thought to be related to ISL-TP accumulation, leading to cell growth inhibition. In vitro, ISL inhibited the growth of PBMCs and human B lymphoblastoid TK6 cells in a concentration-dependent manner, possibly through inhibition of DNA polymerase alpha [8]. At high doses, ISL also inhibited T- and B-cell activation [8]. Clinical and pharmacokinetic modeling have established an ISL-TP threshold of ~1 pmol/10^6^ PBMCs below which reductions in lymphocytes are not anticipated [9,10]. As of today, only two dosing regimens remain under clinical investigation: 0.25 mg daily and 2 mg weekly, as both have shown no clinically significant impact on lymphocyte counts [6,11].

The development of preclinical models that can predict lymphopenia induced by ISL or other NRTTIs may help identify safe and effective doses before clinical development. Here, we investigated whether ISL-induced lymphopenia could be replicated in a macaque model. We selected an oral dose of ISL that results in ISL-TP concentrations in PBMCs that fall within the range observed in humans treated with the monthly 60 mg PrEP dose. To adjust for the shorter half-life of ISL-TP in macaques compared to humans (~50 h and ~190 h, respectively), we performed weekly cycles of treatment that capture C_max_ and C_trough_ values seen with the monthly 60 mg PrEP dose [7]. We demonstrate significant reductions in total lymphocytes and monocytes, CD8 T-cells, and B-cells, and uncover alterations in key gene expression pathways associated with immune regulation, cell proliferation, and inflammation.

## 2. Materials and Methods

### 2.1. Macaques and Drug Administration

Pig-tailed macaques (*Macaca nemestrina*) were housed in a facility that is US Department of Agriculture registered, Office of Laboratory Animal Welfare assured, and Association for Assessment and Accreditation of Laboratory Animal Care accredited. Veterinarians at the Centers for Disease Control and Prevention (CDC) provided husbandry and enrichment care. All macaque procedures were performed under a CDC Institutional Animal Care and Use Committee-approved protocol in accordance with the Guide for the Care and Use of Laboratory Animals [12].

### 2.2. Study Design

Macaques (*n* = 5) received oral ISL (30 mg/kg; WuXi AppTec, Shanghai, China) by gavage under anesthesia once a week for 12 consecutive weeks. The dose of ISL was determined based on a pilot pharmacokinetic study that showed that 30 mg/kg resulted in an intracellular concentration of ISL-triphosphate that is within the range of those seen in humans treated with 60 mg of ISL. Blood was collected once weekly prior to ISL treatment (6–18 weeks per animal; 78 observations) at 0.5, 2, and 24 h after the first dose for pharmacokinetic analysis only; immediately before each weekly dose (12 weeks per animal; 60 observations); and weekly after treatment discontinuation (for 4–6 weeks; 25 observations). Blood volumes were kept to the minimum necessary and never exceeded the CDC IACUC limit on blood collection volumes based on animal weight. Blood was collected in Vacutainer^®^ EDTA Tubes for complete blood counts and in Vacutainer Cell Preparation Tubes (CPT™) for pharmacokinetic analyses and flow cytometry (Becton-Dickson, Franklin Lakes, NJ, USA). PBMCs were isolated from CPTs following the manufacturer’s protocol, treated with BioLegend red blood cell lysis buffer (BioLegend, San Diego, CA, USA), and then counted using trypan blue exclusion on the Countess II FL automated cell counter (Invitrogen, Carlsbad, CA, USA). Plasma aliquots of 500 µL were immediately frozen at −70 °C.

### 2.3. Complete Blood Count

EDTA whole blood samples were inverted 8 times immediately before analysis. Complete blood counts were performed with the Beckman Coulter AcT diff2 Hematology Analyzer (Brea, CA, USA) in the “closed vial whole blood” mode. Hematology parameters analyzed included lymphocytes, monocytes, and granulocytes from blood prior to ISL treatment (6–18 weeks per animal; 78 observations); immediately before each weekly dose (12 weeks per animal; 60 observations); and weekly after treatment discontinuation (for 4–6 weeks; 25 observations).

### 2.4. Multicolor Flow Cytometry and Absolute Counts

Whole blood collected in CPTs was centrifuged for 1 h at 1600× *g* within 2 h of collection. Plasma was removed, and the PBMC layer was collected and washed in phosphate-buffered saline (PBS). The cell pellets were vortexed, then lysed in 3 mL ACK lysis solution for 5 min. PBS was added, up to 14 mL, to stop the lysis reaction, then centrifuged for 20 min at 200× *g* and resuspended in PBS. Cell concentration and viability were determined by using a Countess Cell Counter (Invitrogen). One million cells were aliquoted into individual 5 mL round-bottom Falcon tubes (Corning, NY, USA), pelleted by centrifugation, and resuspended at a concentration of 10 × 10^6^ cells/mL in BD Stain Buffer. Cells were stained for 30 min in the dark with a panel of antibodies according to the manufacturer’s instructions. The specific antibodies used are described in Supplementary Table S1.

After incubation, the samples were washed with 2 mL Stain Buffer, then fixed by using BD Cytofix according to the manufacturer’s protocol, and 100,000 events were acquired on BD FACSymphony A3 flow cytometer (Franklin Lakes, NJ. USA). BD CS&T beads were used to monitor the instrument’s performance and quality control. Stained and fixed cells were analyzed by flow cytometry within 12 h. Data were analyzed with FlowJo v10 software. White blood cells were identified by using side scatter and CD45 gating, followed by double exclusion. Lymphocytes within that gate were isolated by using forward scatter and side scatter, excluding debris and dead cells. To identify distinct populations of T and B lymphocytes, a dot plot of CD3 versus CD20 was created. T-cells were separated further to identify CD4 and CD8 T-cell subsets. The specific gating strategy is shown in Supplementary Figure S1. Fluorescence minus one (FMO) controls were used to identify positive cell populations and to determine the appropriate gating strategy.

Absolute counts were determined using the dual platform method [13]. This involved calculating the absolute count by multiplying the lymphocyte absolute count, derived from a complete blood count (CBC) performed on the Beckman Coulter AcT Diff 2 hematology analyzer, by the percentages of specific lymphocytes and lymphocyte subsets obtained from the BD FACSymphony A3 flow cytometer. Flow analysis was conducted once weekly prior to ISL treatment (5–7 weeks per animal; 30 observations), immediately before each weekly dose (12 weeks per animal; 60 observations), and weekly after treatment discontinuation (for 4–6 weeks; 25 observations).

### 2.5. Measurement of Plasma ISL and Intracellular ISL-TP in PBMCs

ISL in plasma was quantified by liquid chromatography–tandem mass spectrometry (LC-MS/MS) as previously described [13]. The calibrated range for ISL in plasma was 0.1–100 ng/mL. Accuracy and precision in the calibrated range were within 15% and 20% at the lower limit of quantification (LLOQ). Intracellular ISL-TP in PBMCs was also measured as previously described [14]. Briefly, five million live PBMCs were resuspended in 500 µL ice-cold 80% methanol, vortexed for one minute, and immediately frozen at −70 °C. ISL-TP was quantified using previously published LC-MS/MS methods. The calibrated ranges were 0.05–200 for ISL-TP and 0.005–100 ng/mL for dATP. Accuracy and precision were within 20%. Concentrations were normalized to live PBMCs and reported in fmol of ISL-TP/million PBMCs. Pharmacokinetic figures were created by using GraphPad Prism for Windows (version 10.2.3; GraphPad Software, San Diego, CA, USA). Area under the curve values from 2 h to 168 h (AUC_2–168h_) were calculated by using the linear trapezoidal linear interpolation method in Phoenix WinNonlin (version 8.4.0.6172; Certara, Princeton, NJ, USA).

### 2.6. Analysis of Gene Expression in PBMCs

Analysis of gene expression was performed at Emory University EPC Genomics Core. Gene expression patterns were investigated in PBMCs collected immediately before ISL treatment (week 0), after 12 consecutive weeks of ISL treatment (week 12), and 6 weeks after treatment discontinuation (week 18). Briefly, two million PBMCs were lysed with 350 µL of Buffer RLT and then extracted by using the RNeasy Mini kit (Qiagen, Germantown, MD, USA) with on-column DNase digestion. RNA quality was assessed by using a TapeStation 4200 (Agilent, Alpharetta, GA, USA), and then ten nanograms of total RNA were used as input for cDNA synthesis by using the SMART-Seq v4 Ultra Low Input RNA kit (Takara Bio, San Jose, CA, USA) according to the manufacturer’s instructions. Amplified cDNA was fragmented and appended with dual-indexed barcodes by using the Nextera XT DNA Library Preparation kit (Illumina, San Diego, CA, USA). Libraries were validated by capillary electrophoresis on a TapeStation 4200, pooled at equimolar concentrations, and sequenced with PE100 reads on an Illumina NovaSeq 6000, yielding ~23 million reads per sample on average.

Sequencing data were aligned to *Macaca nemestrina* Ensembly release 95 by using STAR 2.7.9a [15]. Transcript abundance estimates were calculated by using the STAR “quantMode GeneCounts” option. DESeq2 was used for transcript normalization and differential expression analysis using the Wald test [16]. Gene set enrichment analysis (GSEA) was performed by using the fGSEA R package (version 1.28.0) with pathway definitions defined in the Hallmark collection of MSigDB [17,18] as well as the C2 canonical KEGG and Reactome pathway sets. Normalized enrichment scores (NES) and adjusted *p* values (*p*_adj_) were used to quantify the degree of enrichment of specific gene sets using the Kolmogorov–Smirnov test. The data can be accessed via GEO (GSE320114).

### 2.7. Statistical Analysis

A repeated measures design was used to compare the effects of ISL on hematology and flow cytometry outcomes across three time periods (pre-treatment, ISL treatment, and post-treatment). All absolute outcome variables were log-transformed to base 10 before analysis. A linear mixed-effects model was fitted with week and period included as fixed effects, while allowing for random intercepts by ID to account for the repeated measures (i.e., multiple observations per animal). Pairwise comparisons between each of the three periods were performed, and the estimates were back-transformed to the original scale [19,20,21]. Since these comparisons are conducted on a log scale and account for differing week distribution across study periods, the resulting estimates reflect multiplicative effects rather than additive ones. *p* values derived from the linear mixed-effects model to assess the statistical significance of the effects were reported utilizing SAS software (version 9.4_M8; SAS Institute Inc., Cary, NC, USA).

## 3. Results

### 3.1. Pharmacokinetic Profile of ISL at First Dose and After Repeated Dosing

**Figure 1:**
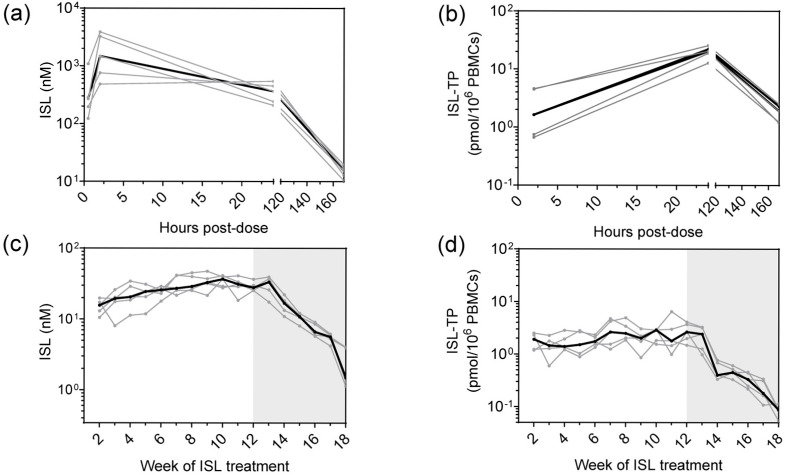
Pharmacokinetic profile of ISL in plasma and peripheral blood mononuclear cells (PBMCs) at first dose and after repeated weekly dosing. Macaques (*n* = 5) received 30 mg/kg of ISL orally once a week for 12 weeks. Each gray line represents log-transformed data for an individual animal; the dark line denotes log-transformed median values. (**a**) Plasma ISL concentrations after the first dose. (**b**) Intracellular ISL-TP concentrations in PBMCs after the first dose. (**c**) Plasma ISL C_trough_ values during weekly ISL treatment (weeks 1–12). (**d**) PBMC ISL-TP C_trough_ values during weekly ISL treatment (weeks 1–12). The shaded area in panels (**c**,**d**) represents the drug washout period (no treatment).

Macaques (*n* = 5) received 30 mg/kg of ISL once weekly for 12 weeks. After the first dose, median (range) ISL concentrations in plasma at 30 min, 2 h, and 24 h were 268 (123–1083), 1476 (482–3862), and 365 (208–546) nM, respectively. ISL was still detected in plasma at day 7 (median = 16 [10–20] nM) (Figure 1a). In PBMCs, the median (range) ISL-TP concentrations at 2 h, 24 h, and 7 days were 1.6 (0.7–4.6), 20.4 (12.8–22.1), and 1.9 (1.2–2.5) pmols/10^6^ cells, respectively (Figure 1b). The median (range) AUC_2–168h_ value for ISL-TP was 1834.0 (1157.4–2351.3) h * pmol/10^6^ PBMCs.

During repeated weekly dosing, the trough concentration (C_trough_) of ISL in plasma ranged between 16 and 37 nM with a median of 27 nM (Figure 1c). Likewise, ISL-TP C_trough_ in PBMCs ranged between 1.4 and 2.9 pmols/10^6^ PBMCs (median = 2.0 pmols/10^6^ PBMCs) (Figure 1d). After the last dose, ISL-TP concentrations showed a biphasic decline characterized by a rapid fall within a week, followed by a more gradual subsequent decline (Supplementary Figure S2).

### 3.2. Effect of ISL on Lymphocytes, Monocytes and Granulocytes

**Figure 2:**
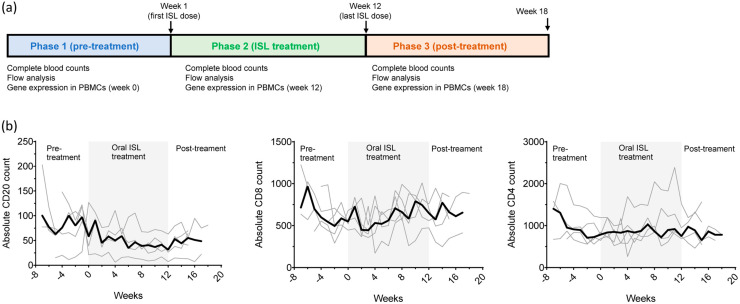
Study design and longitudinal flow cytometry analyses. (**a**) The study was divided in three phases: (i) pre-treatment which included weekly blood collection to define normal complete blood counts and lymphocytes subpopulations for each animal, (ii) ISL treatment which consisted of weekly treatment with 30 mg/kg of oral ISL for 12 weeks, and (iii) post-treatment which followed animals 4–6 weeks after the last ISL dose. PBMCs collected before the first ISL dose at week 1, after 12 weeks of treatment (week 12), and 4 weeks after treatment discontinuation (week 16) were used for gene expression analysis. (**b**) The longitudinal CD20, CD8, and CD4 absolute cell counts derived from flow cytometry analyses for each animal are shown as gray lines. The dark line denoted median values.

We next investigated the effect of ISL on blood cells. The study included a pre-treatment phase to define baseline values, a 12-week treatment period with weekly doses of ISL at 30 mg/kg, and a drug washout phase (Figure 2a). We performed a median of 16 complete blood count measurements (range = 6–18) per animal prior to treatment, weekly measurements during treatment, and a median of six weekly measurements (range = 4–6) following treatment discontinuation.

Treatment with ISL was associated with a 11.9% and 22.4% reduction in total lymphocytes and monocytes from baseline (*p* = 0.0015 and *p* = 0.0003, respectively) (Table 1). After treatment cessation, lymphocytes and monocytes increased by 14.9% (*p* = 0.015) and 38% (*p* = 0.0008), respectively, returning to pre-treatment values. These increases coincided with a biphasic decline in ISL-TP concentrations, decreasing from 2.4 to 0.087 pmol/10^6^ PBMCs over the 35 days after the last ISL dose. (Table 1 and Supplementary Figure S2). ISL treatment was not associated with significant changes in granulocytes (Table 1).

The effect of ISL on lymphocyte subpopulations of CD4, CD8, and CD20 cells was also investigated by flow cytometry prior to ISL treatment (5–7 weeks per animal; 30 observations), immediately before each weekly dose (12 weeks per animal; 60 observations), and weekly after treatment discontinuation (for 4–6 weeks; 25 observations) (Figure 2b). Table 2 shows that 12 weeks of ISL treatment resulted in a significant reduction in absolute CD8 (−18.4%; *p* = 0.0364) and CD20 (−35.3%; *p* = 0.0002) cells but not CD4 cells (−7.4%; *p* = 0.3470). The reduction in CD20 cells was maintained after treatment discontinuation (−50.6%; *p* < 0.0001).

### 3.3. Changes in Gene Expression During ISL Treatment

**Figure 3:**
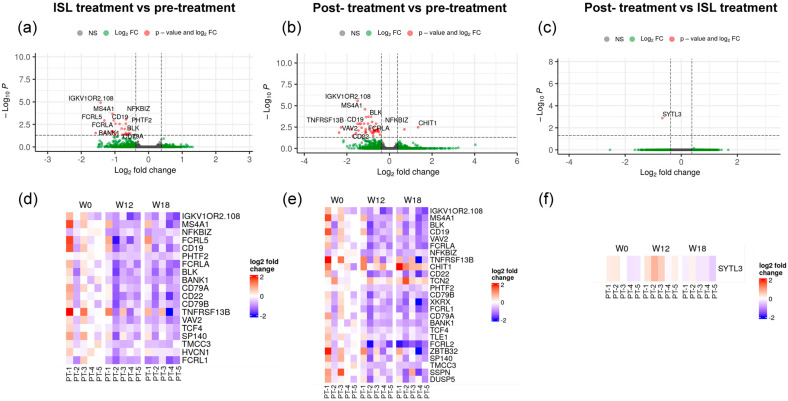
Gene expression analysis in PBMCs during islatravir (ISL) treatment. PBMCs were collected at week 0 (pre-treatment), week 12 (during ISL treatment), and week 18 (6 weeks post-treatment), and RNA-seq was performed. Differential expression analysis was conducted using DESeq2. Panels (**a**–**c**) show volcano plots for (**a**) week 12 vs. week 0, (**b**) week 18 vs. week 0, and (**c**) week 18 vs. week 12. The x-axis shows log2 fold change (log2FC), and the y-axis shows −log10 adjusted *p* value (Wald test). Dashed vertical lines indicate |log2FC| ≥ 1 and the horizontal dashed line indicates adjusted *p* < 0.05. Genes meeting both thresholds are shown in red; genes meeting only the fold-change threshold are shown in green; non-significant genes are shown in gray. Panels (**d**–**f**) show heatmaps of the top differentially expressed genes for the same comparisons. Expression values are displayed as log2 fold change relative to the median pre-treatment (week 0) expression for each gene. Data are shown for individual animals (PT1–PT5). Color scale represents log2 fold change (red, increased; blue, decreased).

Gene expression patterns were investigated in PBMCs collected immediately before treatment, at week 12 of treatment, and at week 18 (6 weeks after treatment discontinuation). The volcano plots and corresponding heatmaps representing genes differentially expressed within study periods are shown in Figure 3.

ISL treatment was associated with underexpression of genes encoding proteins associated with B-cell function (*MS4A1*, *CD19*, *FCRL5*, *CD19*, *FCRLA*, *BLK*, *BANK1*, *CD79A* and *CD79B*), tumor necrosis factor receptor (*TNFRSF13B*), genes predicted to enable CD4 receptor binding activity (*CD22*), genes involved in the regulation of NF-kappa-B transcription factor (*NFKBIZ*), genes associated with cancer cell growth (*TMCC3* and *FCRL1*), and other genes expressed in immune cells, such as voltage-gated hydrogen channel 1 (*HVCN1*), SP140 Nuclear Body Protein (*SP140*), and Immunoglobulin kappa variable 1/OR2-108 (*IGKV1OR2-108*) (Figure 3a,d). The name and description of the genes, along with the log2 fold change in expression and corresponding adjusted *p* values, are shown in Table 3 and Supplementary Table S2. Underexpression of these genes was mostly maintained 6 weeks after treatment discontinuation, with some additional genes being underexpressed, including genes encoding Fc receptor-like 2 (*FCRL2*) and CD300 Antigen-Like Family Member H (*CD300H*) proteins (Figure 3b,e). Little or no changes in gene expression were seen between week 12 of treatment and post-treatment (week 18) (Figure 3c,f). The entire list of genes analyzed, along with the log2 fold change and adjusted *p* values, is shown in Supplementary Files S1–S3.

**Figure 4:**
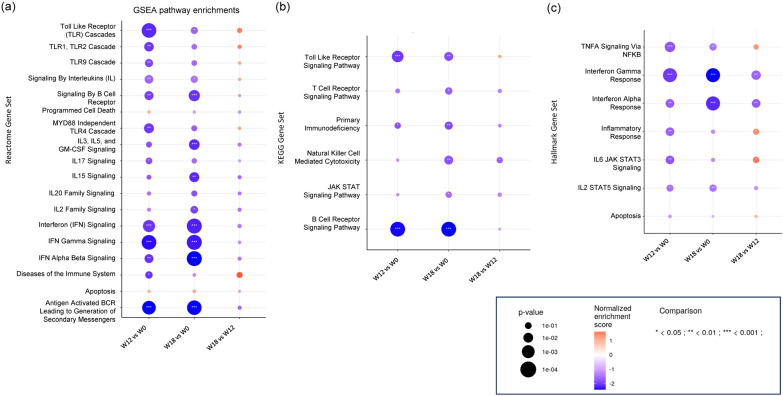
Changes in gene sets associated with ISL treatment. Panels represent the results obtained by GSEA using predefined gene sets from the (**a**) Reactome, (**b**) Kegg, and (**c**) Hallmark collection from MSigDB. Week 0 represents a pre-treatment sample, week 12 represents a treatment sample (last ISL dose), and week 18 represents a post-treatment sample collected 6 weeks after treatment cessation. Enrichment scores and *p* values (Kolmogorov–Smirnov test) are shown for each gene pathway with a figure legend for all panels at the bottom.

We next used GSEA to identify specific gene pathways affected by ISL treatment. Analysis with the Reactome dataset showed negative enrichment of multiple gene pathways associated with immune responses, including IFN signaling (Normalized enrichment score (NES) = −2.0; *p*_adj_ = 0.0001), signaling by interleukins (NES = −1.5, *p*_adj_ = 0.0045), B-cell receptor signaling (NES = −1.9; *p*_adj_ = 0.003), toll-like receptor cascades (NES = −2.0; *p*_adj_ < 0.0001), interferon gamma signaling (NES = −2.21; *p*_adj_ < 0.0001), interferon alpha beta signaling (NES = −2.0; *p*_adj_ < 0.0001), and diseases of the immune system (NES = −2.0; *p*_adj_ = 0.0004) (Figure 4a). Likewise, analysis using the KEGG dataset showed negative enrichment of gene pathways associated with B-cell receptor signaling (NES = −2.3; *p*_adj_ < 0.0001), toll-like receptor signaling (NES = −2.0; *p*_adj_ < 0.0002), and primary immunodeficiency (NES = −1.8; *p*_adj_ = 0.029) (Figure 4b). The Hallmark dataset showed negative enrichment of gene pathways associated with interferon alpha and gamma responses (NES = −1.7; *p*_adj_ = 0.0023 and NES = −2.1; *p*_adj_ < 0.0001, respectively), inflammatory responses (NES = −1.7; *p*_adj_ = 0.0023), IL6 JAK STAT3 signaling (NES = −1.8; *p*_adj_ = 0.0024), TNF alpha signaling via NFKB (NES = −1.8; *p*_adj_ = 0.0004), and ISL2 STAT5 signaling (NES = −1.5; *p*_adj_ = 0.0127) (Figure 4c). Overall, the effect of ISL on gene pathways was maintained after treatment discontinuation (week 18 vs. pre-treatment) with little or no change between treatment and post-treatment samples (week 18 vs. week 12). The entire list of pathways analyzed along the corresponding NES and adjusted *p* values are shown as Supplementary Files (Supplementary Files S4–S6).

## 4. Discussion

We investigated whether macaques could provide a good animal model to investigate ISL-induced lymphopenia. To better model treatment in humans, the dose of ISL was adjusted by allometric scaling to capture C_max_ and C_trough_ ISL-TP values seen with a human monthly dose that reduces total lymphocytes by 10–20%. Treatment was continued for 3 months, which in humans is sufficient to cause lymphopenia. Macaques received ISL once a week instead of monthly to account for the shorter half-life of ISL-TP in macaque PBMCs relative to humans. Under these conditions, ISL-TP C_trough_ levels were above the threshold for lymphopenia in humans and within the range of the monthly 60 mg PrEP dose. With all these pharmacological adjustments, we were able to detect significant reductions in total lymphocytes and monocytes that, as in humans, were fully reversible after treatment cessation. These results underscore the value of the macaque model to study lymphopenia at the preclinical stage of drug development.

As in humans, we found that ISL treatment was associated with significant reductions in CD8 T-cells and B-cells. However, ISL had no effect on CD4 T-cells, with only a small but not significant reduction in absolute CD4 T-cells that was fully reverted after treatment discontinuation. In adults with HIV, treatment with ISL in combination with doravirine or ulonivirine has been associated with dose-dependent decreases in CD4 T-cells [3]. In adults without HIV, monthly doses of ISL are also associated with lymphopenia, although these studies did not specifically assess the effect of ISL on CD4 T-cells [7]. The lack of a significant reduction in CD4 T-cells by ISL in macaques might be explained by a low sample size, a treatment duration that was insufficient to elicit measurable changes, a shorter ISL-TP half-life in macaques compared to humans, or species-specific differences in the mechanisms underlying ISL-induced lymphopenia.

The analysis of gene expression patterns during ISL treatment suggests a broad effect of ISL on immune regulation, cell proliferation, and inflammation. We document underexpression of multiple genes critical for B-cell development, differentiation, signaling, and antibody production. MS4A1 encodes a B-lymphocyte surface molecule that plays a key role in the development and differentiation of B-cells into plasma cells [22]. CD19 plays multiple roles in the activation of B-cells, is critical to mount an optimal immune response, and plays a crucial role in B-cell receptor signaling, development, and differentiation [23]. Members of the Fc receptor-like (FCRL) family are preferentially expressed by B-cells and have a key role in adaptive B-cell signaling [24]. The BLK gene encodes a nonreceptor tyrosine-kinase that is involved in B-lymphocyte development, differentiation, and signaling [25]. *CD79A* and *CD79B* encode the Ig-alpha and Ig-beta proteins of the B-cell antigen receptor, and *CD22* is involved in B-cell activation and negative regulation of the B-cell receptor signaling pathway (more information available at the Human Gene Database, https://www.genecards.org/).

In addition to genes associated with B-cell function, we found underexpression of genes associated with inflammation and cell proliferation. *SP140* is a transcriptional repressor that is essential for macrophage and possibly T-cell function, and loss-of-function mutations are associated with immune disorders, such as multiple sclerosis and B-cell cancers [26]. *NFKBIZ* is a key gene in the regulation of a variety of inflammatory factors in the NF-KB pathway, and *ZBTB32* is required for the regulation of CD8 T-cell responses and is critical for NK cell proliferation, T-cell development, and survival of mature T-cells [27,28,29]. Altogether, our findings highlight alterations in key gene expression pathways associated with immune regulation, cell proliferation, and inflammation. However, underexpression of genes could also reflect changes in the relative abundance of cell types, particularly B-cells. Thus, additional cell-type deconvolution analysis is needed to determine transcriptional changes within the remaining B-cell population. These studies will provide a better understanding of gene expression differences within cell populations as well as potential correlations between transcriptomic changes and cell function.

Our study has some potential limitations. First, we did not have an untreated control group to definitively attribute lymphopenia to ISL treatment. However, we mitigated this limitation by incorporating repeated baseline measurements, which allowed us to characterize normal fluctuations in lymphocyte counts and more accurately assess treatment-related changes in individual animals. Notably, total lymphocyte counts returned to baseline levels following cessation of ISL treatment, supporting a treatment-related effect. Second, the study was limited to a sample size of five animals per group, with unequal numbers of weeks across the baseline, treatment, and post-treatment periods. This imbalance may introduce variability that could affect the robustness of the results. Although a linear mixed-effects model was applied to account for repeated measures, the differences in the number of observations across treatment periods may reduce the statistical power of the pairwise comparisons. For instance, the number of follow-up samples was limited to only 25, which might add uncertainty about the rate of cell recovery after ISL discontinuation. Therefore, while the model appropriately handles within-animal correlation, future studies should aim for more balanced numbers of observations to enhance the generalizability of the findings. Further validation in larger cohorts is thus warranted.

In summary, we demonstrate that high doses of oral ISL induce lymphopenia in macaques, reproducing clinical toxicity seen in humans. Our study underscores the utility of the macaque model to investigate immune alterations at the preclinical stage. These studies may be particularly useful for evaluating the safety of other NRTTIs currently in clinical development, such as MK8527. Demonstrating safety preclinically in the macaque model might also inform maximum dose selection over long dosing intervals exceeding one month prior to human trials.

## Figures and Tables

**Table 1:** Pairwise comparison of total lymphocytes, monocytes, and granulocytes by study periods (pre-treatment, ISL treatment, and post-treatment) after adjusting for week using a linear mixed-effects model.

Comparison	Estimate (95% CI)	% Change	*p* Value
Lymphocytes ISL treatment vs. pre-treatment Post-treatment vs. ISL treatment Post-treatment vs. pre-treatment	0.880 (0.815, 0.952) 1.149 (1.034, 1.278) 1.011 (0.910, 1.125)	−11.9 14.9 1.2	0.0015 0.0105 0.8244
Monocytes ISL treatment vs. pre-treatment Post-treatment vs. ISL treatment Post-treatment vs. pre-treatment	0.776 (0.677, 0.889) 1.381 (1.147, 1.664) 1.072 (0.890, 1.291)	−22.4 38 7.2	0.0003 0.0008 0.4620
Granulocytes ISL treatment vs. pre-treatment Post-treatment vs. ISL treatment Post-treatment vs. pre-treatment	0.997 (0.818, 1.215) 0.952 (0.727, 1.247) 0.949 (0.725, 1.243)	−0.3 −4.5 −5.1	0.9781 0.7193 0.7038

Outcomes were log-transformed to base 10 and back-transformed to original scale; *p* values represent pairwise comparison significance.

**Table 2:** Pairwise comparison of absolute CD4, CD8, and CD20 cell counts by study periods (pre-treatment, ISL treatment, and post-treatment) after adjusting for week using a linear mixed-effects model.

Comparison	Estimate (95% CI)	% Change	*p* Value
CD4+ cells ISL treatment vs. pre-treatment Post-treatment vs. ISL treatment Post-treatment vs. pre-treatment	0.926 (0.788, 1.088) 1.081(0.968, 1.210) 1.001 (0.837, 1.198)	−7.4 8.1 0.1	0.3470 0.1653 0.9891
CD8+ cells ISL treatment vs. pre-treatment Post-treatment vs. ISL treatment Post-treatment vs. pre-treatment	0.816 (0.675, 0.987) 1.153 (1.012, 1.314) 0.941 (0.762, 1.162)	−18.4 15.3 −5.9	0.0364 0.0329 0.5684
CD20+ cells ISL treatment vs. pre-treatment Post-treatment vs. ISL treatment Post-treatment vs. pre-treatment	0.647 (0.517, 0.809) 0.506 (0.394, 0.649) 0.783 (0.671, 0.913)	−35.3 −21.7 −50.6	0.0002 <0.0001 0.0021

Outcomes were log-transformed to base 10 and back-transformed to original scale; *p* values represent pairwise comparison significance.

**Table 3:** Differentially expressed genes observed during ISL treatment relative to pre-treatment.

Name/Code	Description	Base Mean Expression	Log2 Fold Change	Fold Change Standard Error	Adjusted *p* Value
IGKV1OR2-108	Immunoglobulin kappa variable 1/OR2-108	85	−1.43	0.23	0.000012
MS4A1	Membrane Spanning 4-Domains A1	398	−1.07	0.19	0.0002
NFKBIZ	NF-kappa-B inhibitor zeta	992	−0.67	0.13	0.00087
FCRL5	Fc receptor-like 5	64	−1.31	0.26	0.0011
CD19	Differentiation Antigen CD19	236	−1.03	0.2	0.0011
PHTF2	Putative homeodomain transcription factor 2	332	−0.68	0.14	0.0027
FCRLA	Fc receptor-like A	180	−0.99	0.2	0.0027
BLK	BLK Proto-Oncogene, Src Family Tyrosine Kinase	178	−0.86	0.18	0.0028
BANK1	B-cell scaffold protein with ankyrin repeats 1	196	−0.8	0.18	0.0091
MEF2C	Myocyte Enhancer Factor 2C	479	−0.55	0.12	0.0093
CD79A	B-Cell Antigen Receptor Complex-Associated Protein Alpha Chain	1198	−0.71	0.16	0.01
ENSMNEG00000021731	Ig-like domain-containing protein	82	−1.07	0.25	0.018
CD22	CD22 molecule	72	−1.06	0.25	0.021
ENSMNEG00000005794	Ig-like domain-containing protein	46	−1.31	0.31	0.029
ENSMNEG00000037499	Ig-like domain-containing protein	22	−1.57	0.38	0.029
ASPH	Aspartate Beta-Hydroxylase	329	−0.55	0.13	0.029
CD79B	B-Cell Antigen Receptor Complex-Associated Protein Beta Chain	784	−0.67	0.16	0.029
TNFRSF13B	Tumor necrosis factor receptor superfamily member 13B	70	−1.18	0.29	0.029
VAV2	Vav Guanine Nucleotide Exchange Factor 2	69	−0.97	0.24	0.029
TCF4	Transcription Factor 4 or Immunoglobulin Transcription Factor 2	251	−0.59	0.14	0.032
SP140	SP140 Nuclear Body Protein	76	−0.78	0.2	0.044
TMCC3	Transmembrane And Coiled-Coil Domain Family 3	99	−0.74	0.19	0.044
HVCN1	Hydrogen Voltage-Gated Channel 1	343	−0.65	0.17	0.044
ENSMNEG00000017115	Ig-like domain-containing protein	2342	−0.63	0.16	0.044

## Data Availability

The original contributions presented in this study are included in the article/Supplementary Material. Further inquiries can be directed to the corresponding author.

## Supplementary Materials

The following supporting information can be downloaded at: https://www.mdpi.com/article/10.3390/pharmaceutics18030381/s1, Figure S1: Gating strategy used to analyze CD4, CD8, and CD20 cells. Figure S2: Plasma ISL (a) and intracellular ISL-TP (b) concentrations after the last dose. Table S1: Antibodies used for flow cytometry. Table S2: Gene cards for differentially expressed genes following ISL treatment. File S1: Differentially expressed genes (Week 12 vs pretreatment). File S2: Differentially expressed genes (Week 18 vs pretreatment). File S3: Differentially expressed genes (Week 18 vs week 12). File S4: HALLMARK_PBMC_GSEA. File S5: KEGG_PBMC_GSEA. File S6: REACTOME_PBMC_GSEA.
