# Supplementary material for: Oral Islatravir in Macaques Decreases Lymphocytes and Monocytes and Is Associated with Immune Alterations
Source: Pharmaceutics. 2026 Mar 20;18(3):381. doi: 10.3390/pharmaceutics18030381 (PMC13030831; doi:10.3390/pharmaceutics18030381)
Supplement: Supplementary file 1 [file pharmaceutics-18-00381-s001.zip › Supp. Figures and Tables.pdf]

Supplementary materials

Oral Islatravir in Macaques Decreases Lymphocytes and Monocytes and Is Associated with Immune Alterations

Michele B. Daly [1], Daniel Kim [1], Seidu Inusah [2], Dawn Little [1], Jiyoung S. Kim [1], Natalia Makarova [1], Tiancheng E. Edwards [1], James Mitchell [1], Walid Heneine [1], Yi Pan [2], Charles W. Dobard [1] and J. Gerardo García-Lerma [1,*]

[1] Laboratory Branch, Division of HIV Prevention, National Center for HIV, Viral Hepatitis, STD, and TB Prevention, Centers for Disease Control and Prevention, Atlanta, GA 30333, USA; nmj0@cdc.gov (M.B.D.); daniel.kim3@emory.edu (D.K.); dawn.little@emory.edu (D.L.); sammi.kim@emory.edu (J.S.K.); wcn5@cdc.gov (N.M.); obf0@cdc.gov (T.E.E.); zjl9@cdc.gov (J.M.); wmh2@cdc.gov (W.H.); gok5@cdc.gov (C.W.D.)

[2] Quantitative Sciences Branch, Division of HIV Prevention, National Center for HIV, Viral Hepatitis, STD, and TB Prevention, Centers for Disease Control and Prevention, Atlanta, GA 30333, USA;
nby6@cdc.gov (S.I.); yi.pan@bms.com (Y.P.)

* Correspondence: jng5@cdc.gov; Tel.: +1-(404)-639-4987

Running title: Islatravir toxicity in macaques

Supplementary Figures

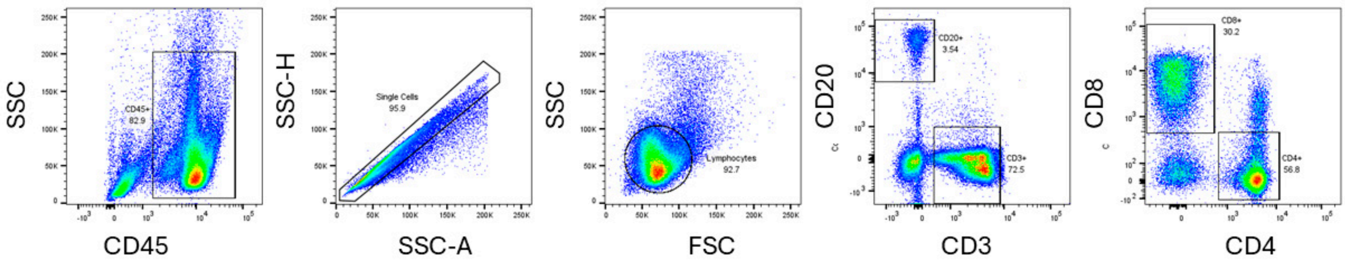

Supplementary Figures S1. Gating strategy used to analyze CD4, CD8, and CD20 cells.

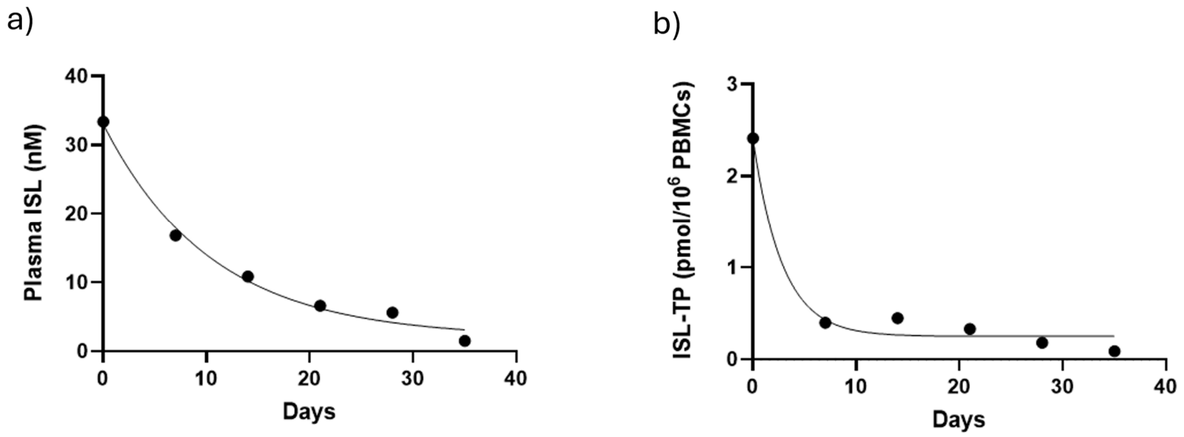

Supplementary Figures S2. Plasma ISL (a) and intracellular ISL-TP (b) concentrations after the last dose. ISL and ISL-TP showed linear and biphasic elimination patterns respectively.

Supplementary Tables

Supplementary Table S1. Antibodies used for flow cytometry

Marker	Clone	Fluorochrome	Company	Catalog #	Cell Type
CD3	SP34-2	Alexa Fluor 700	BD Biosciences	557917	T Cells
CD4	L200	PerCP-Cy 5.5	BD Biosciences	552838	CD4+ T Cells
CD8	RPA-T8	Brilliant Violet 421	BioLegend	301036	CD8+ T Cells
CD20	2H7	Brilliant Violet 650	BioLegend	302336	B Cells
CD45	D058-1283	FITC	BD Biosciences	557803	Leukocytes

Supplementary Table S2. Gene cards for differentially expressed genes following ISL treatment

GENE	GENE DATABASE	FUNCTION
IGKV1OR2	IGKV1OR-2 Gene - GeneCards | IGKV1OR-2 Pseudogene	IGKV1OR-2 (Immunoglobulin Kappa Variable 1/OR-2 (Pseudogene)) is a Pseudogene.
MS4A1	MS4A1 Gene - GeneCards | CD20 Protein | CD20 Antibody	This gene encodes a member of the membrane-spanning 4A gene family. Members of this nascent protein family are characterized by common structural features and similar intron/exon splice boundaries and display unique expression patterns among hematopoietic cells and nonlymphoid tissues. This gene encodes a B-lymphocyte surface molecule which plays a role in the development and differentiation of B-cells into plasma cells. This family member is localized to 11q12, among a cluster of family members.
NFKBIZ	NFKBIZ Gene - GeneCards | IKBZ Protein | IKBZ Antibody	This gene is a member of the ankyrin-repeat family and is induced by lipopolysaccharide (LPS). The C-terminal portion of the encoded product which contains the ankyrin repeats, shares high sequence similarity with the I kappa B family of proteins. The latter are known to play a role in inflammatory responses to LPS by their interaction with NF-B proteins through ankyrin-repeat domains. Studies in mouse indicate that this gene product is one of the nuclear I kappa B proteins and an activator of IL-6 production.
FCRL5	FCRL5 Gene - GeneCards | FCRL5 Protein | FCRL5 Antibody	This gene encodes a member of the immunoglobulin receptor superfamily and the Fc-receptor like family. This gene and several other Fc receptor-like gene members are clustered on the long arm of chromosome 1. The encoded protein is a single-pass type I membrane protein and contains 8 immunoglobulin-like C2-type domains. This gene is implicated in B cell development and lymphomagenesis.
CD19	CD19 Gene - GeneCards | CD19 Protein | CD19 Antibody	This gene encodes a member of the immunoglobulin gene superfamily. Expression of this cell surface protein is restricted to B cell lymphocytes. This protein is a reliable marker for pre-B cells but its expression diminishes during terminal B cell differentiation in antibody secreting plasma cells. The protein has two N-terminal extracellular Ig-like domains separated by a non-Ig-like domain, a hydrophobic transmembrane domain, and a large C-terminal cytoplasmic domain. This protein forms a complex with several membrane proteins including complement receptor type 2 (CD21) and tetraspanin (CD81) and this complex reduces the threshold for antigen-initiated B cell activation. Activation of this B-cell antigen receptor complex activates the phosphatidylinositol 3-kinase signalling pathway and the subsequent release of intracellular stores of calcium ions. This protein is a target of chimeric antigen receptor (CAR) T-cells used in the treatment of lymphoblastic leukemia. Mutations in this gene are associated with the disease common variable immunodeficiency 3 (CVID3) which results in a failure of B-cell

		differentiation and impaired secretion of immunoglobulins. CVID3 is characterized by hypogammaglobulinemia, an inability to mount an antibody response to antigen, and recurrent bacterial infections.
PHTF2	PHTF2 Gene - GeneCards | PHTF2 Protein | PHTF2 Antibody	Located in endoplasmic reticulum
FCRLA	FCRLA Gene - GeneCards | FCRLA Protein | FCRLA Antibody	This gene encodes a protein similar to receptors for the Fc fragment of gamma immunoglobulin (IgG). These receptors, referred to as FCGRs, mediate the destruction of IgG-coated antigens and of cells induced by antibodies. This encoded protein is selectively expressed in B cells, and may be involved in their development. This protein may also be involved in the development of lymphomas.
BLK	BLK Gene - GeneCards | BLK Protein | BLK Antibody	This gene encodes a nonreceptor tyrosine-kinase of the src family of proto-oncogenes that are typically involved in cell proliferation and differentiation. The protein has a role in B-cell receptor signaling and B-cell development.
BANK1	BANK1 Gene - GeneCards | BANK1 Protein | BANK1 Antibody	The protein encoded by this gene is a B-cell-specific scaffold protein that functions in B-cell receptor-induced calcium mobilization from intracellular stores. This protein can also promote Lyn-mediated tyrosine phosphorylation of inositol 1,4,5-trisphosphate receptors. Polymorphisms in this gene are associated with susceptibility to systemic lupus erythematosus.
CD79A	CD79A Gene - GeneCards | CD79A Protein | CD79A Antibody	The B lymphocyte antigen receptor is a multimeric complex that includes the antigen-specific component, surface immunoglobulin (Ig). Surface Ig non-covalently associates with two other proteins, Ig-alpha and Ig-beta, which are necessary for expression and function of the B-cell antigen receptor. This gene encodes the Ig-alpha protein of the B-cell antigen component.
CD22	CD22 Gene - GeneCards | CD22 Protein | CD22 Antibody	Predicted to enable CD4 receptor binding activity; protein phosphatase binding activity; and sialic acid binding activity. Involved in B cell activation; negative regulation of B cell receptor signaling pathway; and regulation of endocytosis. Located in early endosome and recycling endosome.
CD79B	CD79B Gene - GeneCards | CD79B Protein | CD79B Antibody	The B lymphocyte antigen receptor is a multimeric complex that includes the antigen-specific component, surface immunoglobulin (Ig). Surface Ig non-covalently associates with two other proteins, Ig-alpha and Ig-beta, which are necessary for expression and function of the B-cell antigen receptor. This gene encodes the Ig-beta protein of the B-cell antigen component.
TNFRSF13B	TNFRSF13B Gene - GeneCards | TR13B Protein | TR13B Antibody	The protein encoded by this gene is a lymphocyte-specific member of the tumor necrosis factor (TNF) receptor superfamily. It interacts with calcium-modulator and cyclophilin ligand (CAML). The protein induces activation of the transcription factors NFAT, AP1, and NF-kappa-B and plays a crucial role in humoral immunity by interacting with a TNF ligand.
VAV2	VAV2 Gene - GeneCards | VAV2	VAV2 is the second member of the VAV guanine nucleotide exchange factor family of oncogenes. Unlike VAV1, which is expressed exclusively in hematopoietic cells, VAV2 transcripts

	Protein | VAV2 Antibody	were found in most tissues. Alternatively spliced transcript variants encoding different isoforms have been found for this gene
TCF4	TCF4 Gene - GeneCards | ITF2 Protein | ITF2 Antibody	This gene encodes transcription factor 4, a basic helix-loop-helix transcription factor. The encoded protein recognizes an Ephrussi-box ('E-box') binding site ('CANNTG') - a motif first identified in immunoglobulin enhancers. This gene is broadly expressed, and may play an important role in nervous system development.
SP140	SP140 Gene - GeneCards | SP140 Protein | SP140 Antibody	This gene encodes a member of the SP100 family of proteins, which share common domains including an N-terminal homogeneously staining region domain followed by a SP100/autoimmune regulator/NucP41/P75/deformed epidermal autoregulatory factor domain, a plant homeobox zinc finger, and a bromodomain. The encoded protein is interferon-inducible and is expressed at high levels in the nuclei of leukocytes. Variants of this gene have been associated with multiple sclerosis, Crohn's disease, and chronic lymphocytic leukemia.
TMCC3	TMCC3 Gene - GeneCards | TMCC3 Protein | TMCC3 Antibody	Enables 14-3-3 protein binding activity and identical protein binding activity. Located in endoplasmic reticulum.
HVCN1	HVCN1 Gene - GeneCards | HVCN1 Protein | HVCN1 Antibody	This gene encodes a voltage-gated protein channel protein expressed more highly in certain cells of the immune system. Phagocytic cells produce superoxide anions which require this channel protein, and in B cells this same process facilitates antibody production. This same channel protein, however, can also regulate functions in other cells including spermatozoa.
FCRL1	FCRL1 Gene - GeneCards | FCRL1 Protein | FCRL1 Antibody	This gene encodes a member of the immunoglobulin receptor superfamily and is one of several Fc receptor-like glycoproteins clustered on the long arm of chromosome 1. The encoded protein contains three extracellular C2-like immunoglobulin domains, a transmembrane domain and a cytoplasmic domain with two immunoreceptor-tyrosine activation motifs. This protein may play a role in the regulation of cancer cell growth.
CHIT1	CHIT1 Gene - GeneCards | CHIT1 Protein | CHIT1 Antibody	Chitotriosidase is secreted by activated human macrophages and is markedly elevated in plasma of Gaucher disease patients. The expression of chitotriosidase occurs only at a late stage of differentiation of monocytes to activated macrophages in culture. Human macrophages can synthesize a functional chitotriosidase, a highly conserved enzyme with a strongly regulated expression. This enzyme may play a role in the degradation of chitin-containing pathogens.
TCN2	TCN2 Gene - GeneCards | TCO2 Protein | TCO2 Antibody	This gene encodes a member of the vitamin B12-binding protein family. This family of proteins, alternatively referred to as R binders, is expressed in various tissues and secretions. This plasma protein binds cobalamin and mediates the transport of cobalamin into cells. This protein and other mammalian cobalamin-binding proteins, such as transcobalamin I and gastric intrisic factor, may have evolved by duplication of a common ancestral gene.

XKRX	XKRX Gene - GeneCards | XKR2 Protein | XKR2 Antibody	This gene encodes a protein that is related to a component of the XK/Kell complex of the Kell blood group system. The encoded protein includes several transmembrane domains, is known to be exposed to the cell surface, and may function as a membrane transporter.
TLE1	TLE1 Gene - GeneCards | TLE1 Protein | TLE1 Antibody	Enables identical protein binding activity and transcription corepressor activity. Involved in negative regulation of I-kappaB kinase/NF-kappaB signaling; negative regulation of anoikis; and regulation of gene expression. Located in cytosol and nucleoplasm. Part of beta-catenin-TCF complex.
FCRL2	FCRL2 Gene - GeneCards | FCRL2 Protein | FCRL2 Antibody	This gene encodes a member of the immunoglobulin receptor superfamily and is one of several Fc receptor-like glycoproteins clustered on the long arm of chromosome 1. The encoded protein has four extracellular C2-type immunoglobulin domains, a transmembrane domain and a cytoplasmic domain that contains one immunoreceptor-tyrosine activation motif and two immunoreceptor-tyrosine inhibitory motifs. This protein may be a prognostic marker for chronic lymphocytic leukemia.
ZBTB32	ZBTB32 Gene - GeneCards | ZBT32 Protein | ZBT32 Antibody	Enables DNA-binding transcription repressor activity, RNA polymerase II-specific; identical protein binding activity; and sequence-specific double-stranded DNA binding activity. Involved in negative regulation of transcription by RNA polymerase II. Located in nucleus.
SSPN	SSPN Gene - GeneCards | SSPN Protein | SSPN Antibody	This gene encodes a member of the dystrophin-glycoprotein complex (DGC). The DGC spans the sarcolemma and is comprised of dystrophin, syntrophin, alpha- and beta-dystroglycans and sarcoglycans. The DGC provides a structural link between the subsarcolemmal cytoskeleton and the extracellular matrix of muscle cells.
DUSP5	DUSP5 Gene - GeneCards | DUS5 Protein | DUS5 Antibody	The protein encoded by this gene is a member of the dual specificity protein phosphatase subfamily. These phosphatases inactivate their target kinases by dephosphorylating both the phosphoserine/threonine and phosphotyrosine residues. They negatively regulate members of the mitogen-activated protein (MAP) kinase superfamily (MAPK/ERK, SAPK/JNK, p38), which are associated with cellular proliferation and differentiation. Different members of the family of dual specificity phosphatases show distinct substrate specificities for various MAP kinases, different tissue distribution and subcellular localization, and different modes of inducibility of their expression by extracellular stimuli. This gene product inactivates ERK1, is expressed in a variety of tissues with the highest levels in pancreas and brain, and is localized in the nucleus
SUTL3	SYTL3 Gene - GeneCards | SYTL3 Protein | SYTL3 Antibody	The protein encoded by this gene belongs to a family of peripheral membrane proteins that play a role in vesicular trafficking. This protein binds phospholipids in the presence of calcium ions.
